# Supplementary material for: Functional diversity in human song
Source: PLoS One. 2024 Jul 12;19(7):e0307032. doi: 10.1371/journal.pone.0307032 (PMC11244838; doi:10.1371/journal.pone.0307032)
Supplement: S1 File — (PDF) [file pone.0307032.s001.pdf]

Journal: PLOS ONE

Supporting information for: Functional diversity in human song

Authors: Lucas Colares[1*], Ray Balieiro Lopes-Neto[2], Alexandre Sampaio de Siqueira[3], Camila Ferreira Leão[4], Arianne Flexa de Castro[4], Bárbara Dunck[4,5].

Affiliations: [1] Programa de Pós-Graduação em Biodiversidade Animal, Laboratório de Ecologia Teórica e Aplicada, Universidade Federal de Santa Maria, Santa Maria – RS, Brazil. [2] Programa de Pós-Graduação em Botânica Tropical, Museu Paraense Emílio Goeldi, Belém – PA, Brazil. [3] Programa de Pós-Graduação em Ecologia e Recursos Naturais, Universidade Federal de São Carlos, São Carlos – SP, Brazil. [4] Programa de Pós-Graduação em Ecologia, Universidade Federal do Pará, Belém – PA, Brazil. [5] Instituto Socioambiental e dos Recursos Hídricos, Universidade Federal Rural da Amazônia, Belém – PA, Brazil.

* **Correspondent author:** Lucas Colares (lucasfcolares@gmail.com)

This PDF file includes:

S1 Table – List of Genius tags for musical genres (page 2).
S2 Table – Correlation matrix between trait at the level of artists (page 10).
S3 Table – Correlation matrix between trait at the level of albums (page 11).
S4 Table – Correlation matrix between trait at the level of tracks (page 12).
S5 Table – Variable loadings from the Principal Component Analysis (page 13).
S6 Table – Selection steps for functional diversity models (page 14).
S7 Table – Coefficients of models in which functional diversity is the predictor (page 15).
S8 Table – Selection steps for models in which traits are the predictors (page 16).
S9 Table – Coefficients of models in which traits are the predictors (page 17).
S10 Table – Selection steps for models in which traits are the predictors per musical genre (page 19).
S11 Table – Coefficients of models in which traits are the predictors separated by the seven major musical genres (page 23).
S1 Figure – Distribution of continuous traits (page 27).
S2 Figure – Frequency of categorical traits (page 28).
S3 Figure – Associations between popularity and traits per musical genre (page 29).
S4 Figure – Unidimensional functional space of artists (page 30).
S5 Figure – Association between year of career launch and popularity (page 32).

Supporting tables

Table S1. List of Genius tags for musical genres. A list of all Genius tags that were retrieved during the study and in how many songs they appeared on (N). Tags that do not represented a musical genre were excluded from the dataset; these are marked as "*Removed*". All tags that represented a musical genre were included in the final dataset and are marked as "*Included*", but only tags that appeared in more than 500 of all 10,444 songs remained for the data analysis, these are highlighted in bold.

Tag	N	Action
A Cappella	87	Removed
Abstract Rap	4	Included
Acoustic	115	Removed
Adult Alternative	170	Included
Adult Contemporary	255	Included
Afrobeats	14	Included
Album-Oriented Rock (AOR)	12	Removed
Alternative	497	Included
Alternative Country	43	Included
Alternative Dance	26	Included
Alternative Metal	3	Included
Alternative Pop	441	Included
Alternative R&B	268	Included
Alternative Rock	390	Included
Ambient	27	Included
American Folk	28	Removed
Americana	61	Included
Art	9	Removed
Art Pop	58	Included
Art Rock	12	Included
Atlanta	464	Removed
Australia	212	Removed
Ballad	630	Removed
Baroque Pop	50	Included
Bass Music	3	Included
Bedroom Pop	5	Included
Beef	36	Removed
Blue-Eyed Soul	11	Included
Bluegrass	23	Included
Blues	32	Included
Blues Rock	72	Included

Booklet	31	Removed
Boom Bap	61	Included
Bossa Nova	3	Included
Bounce	20	Included
Boy Band	86	Removed
Breakbeat	3	Included
British Folk	5	Removed
British Rock	278	Removed
Britpop	47	Removed
Bubblegum Pop	70	Included
Canada	483	Removed
Celtic	4	Included
Chamber Music	24	Included
Children's Music	213	Included
Chill	14	Included
Chillout	4	Included
Christian	30	Included
Christian Rap	13	Included
Christmas	193	Removed
Classical Crossover	13	Included
Classical Music	22	Included
Cloud Rap	73	Included
Colombia	7	Removed
Comedy	18	Removed
Conscious Hip Hop	4	Included
Conscious Hip-Hop	144	Included
Contemporary Folk	35	Included
**Country**	**1589**	**Included**
Country Rap	8	Included
Cover	422	Removed
COVID-19	4	Removed
Crunk	18	Included
Cuba	36	Removed
Dance	277	Included
Dance Pop	32	Included
Dancehall	69	Included
Dance-Pop	276	Included
Dark Ambient	5	Included
Dark Cabaret	5	Included
Dark Pop	81	Included
Deep House	22	Included

Demo	16	Removed
Dirty South	72	Removed
Disco	20	Included
Disney	12	Removed
Diss Track	5	Included
DMV	250	Removed
Doo-Wop	15	Included
Downtempo	13	Included
Dream Pop	34	Included
Drill	7	Included
Drum & Bass	6	Included
Dubstep	20	Included
East Coast	271	Removed
Easy Listening	110	Removed
EDM	51	Included
Eighties	157	Removed
Electro	47	Included
Electro Hop	2	Included
Electro House	84	Included
Electro Pop	39	Included
Electro-Funk	14	Included
Electro-Hop	27	Included
Electronic	425	Included
Electronic Rock	54	Included
Electronic Trap	20	Included
Electronica	22	Included
Electro-Pop	484	Included
Electro-Soul	3	Included
Em Português	3	Removed
Emo	46	Included
Emo Pop	5	Included
Emo Rap	87	Included
Emo Trap	42	Included
En Español	66	Removed
En Français	3	Removed
Eurodance	22	Removed
Experimental	50	Included
Experimental Hip-Hop	51	Included
Experimental Pop	11	Included
Experimental Rock	8	Included
Feminism	31	Removed

Folk	187	Included
Folk Pop	75	Included
Folk Rock	243	Included
France	18	Removed
Freestyle	21	Included
French Pop	6	Removed
Funk	74	Included
Funk Nacional	1	Included
Funk Rock	16	Included
Funk-Pop	30	Included
Future Bass	18	Included
Future House	20	Included
Gaming	4	Removed
Gangsta Rap	43	Included
G-Funk	12	Included
Glam Metal	5	Included
Glam Rock	25	Included
Glitch Hop	6	Included
Gospel	43	Included
Gothic Rock	3	Included
Grime	3	Included
Grunge	3	Included
Hard Rock	35	Included
Hardcore Hip Hop	6	Included
Hardcore Hip-Hop	159	Included
Heartland Rock	226	Included
Hip Hop	7	Included
Hip-Hop	344	Included
Holiday	78	Removed
Honky Tonk	10	Included
Horrorcore	64	Included
House	94	Included
Hyphy	6	Included
Indie	75	Included
Indie Folk	11	Included
Indie Pop	78	Included
Indie Rap	46	Included
Indie Rock	78	Included
Industrial	17	Included
Industrial Hip-Hop	15	Included
Industrial Rock	4	Included

Instrumental	62	Included
Interlude	79	Removed
Intro	44	Removed
Ireland	245	Removed
Jamaica	27	Removed
Jazz	140	Included
Jazz Fusion	24	Included
Jazz Rap	49	Included
Jazz-Funk	3	Included
Korean	4	Removed
Latin Music	36	Removed
Latin Pop	35	Removed
Latin Trap	7	Included
Latin Urban	18	Removed
Letter	3	Removed
LGBTQ+	31	Removed
Liner Notes	6	Included
Literature	5	Removed
Live	36	Removed
Lo-Fi	24	Included
Lullaby	3	Removed
Marvel	3	Removed
Memes	42	Removed
Memorial	18	Removed
Memphis	7	Removed
Mental Health	8	Removed
Metal	8	Included
México	15	Removed
Modern Classical	4	Included
Motown	32	Removed
Musicals	24	Removed
Neo Soul	83	Included
Neo-Psychedelia	10	Included
New Jack Swing	7	Included
New Orleans R&B	1	Included
New Wave	39	Included
New York	3	Removed
New Zealand	20	Removed
News	3	Removed
Nigeria	11	Removed
Nineties	23	Removed

Non-Music	96	Included
Nu Disco	53	Included
Nu-Metal	3	Included
Orchestral	71	Included
Outlaw Country	8	Included
Outro	10	Removed
Patois	4	Removed
Piano	200	Removed
Poetry	11	Removed
Politics	30	Removed
**Pop**	**4775**	**Included**
Pop Country	332	Included
Pop Rap	50	Included
Pop Rock	2	Included
Pop-Punk	51	Included
**Pop-Rock**	**742**	**Included**
Posse Cut	13	Included
Post-Britpop	35	Removed
Post-Grunge	9	Included
Post-Punk	32	Included
Post-Punk Revival	4	Included
Post-Rock	3	Included
Power Pop	147	Included
Producer	175	Removed
Progressive House	22	Included
Progressive Rock	5	Included
Protest Songs	21	Removed
Psychedelic	46	Included
Psychedelic Rock	16	Included
Psychedelic Soul	3	Included
Puerto Rico	7	Removed
Punk Rock	62	Included
**R&B**	**1784**	**Included**
Race / Ethnicity	3	Removed
Ragga	4	Included
**Rap**	**4070**	**Included**
Rap Rock	63	Included
Reggae	40	Included
Reggae Rock	4	Included
Reggaeton	12	Included
Remix	113	Removed

**Rock**	**2895**	**Included**
Rockabilly	14	Included
Satire	11	Removed
Scandinavia	1	Included
Screamo	3	Included
Screen	9	Removed
Seventies	119	Removed
Shoegaze	4	Included
Singer-Songwriter	1365	Removed
Sixties	19	Removed
Ska	8	Included
Skit	72	Included
Slowcore	3	Included
Smooth Jazz	3	Included
Soft Rock	36	Included
Soul	213	Included
Soul Jazz	4	Included
Soul Pop	139	Included
Soul Rap	1	Included
Soundtrack	146	Removed
South Africa	3	Removed
Southern Rock	28	Removed
Space Rock	3	Included
Spoken Word	59	Included
Sverige	1	Included
Swing	3	Included
Synth Pop	1	Included
Synth Rock	24	Included
Synth-Pop	263	Included
Synthwave	11	Included
Techno	26	Included
Teen Pop	289	Included
Tracklist + Album Art	8	Removed
Trance	7	Included
**Trap**	**1211**	**Included**
Trap Metal	2	Included
Trinidad & Tobago	99	Removed
Trip-Hop	20	Included
Tropical House	33	Included
UK	704	Removed
UK R&B	21	Removed

UK Rap	17	Removed
Underground Hip-Hop	12	Included
Viral Videos	8	Removed
West Coast	171	Removed
Yacht Rock	3	Removed

Table S2. Correlation matrix between trait at the level of artists. Correlation matrix between all traits at the artist level (i.e., mean trait values considering all songs from an artist). Values highlighted in bold represent correlation between two traits higher than the threshold of |0.6| adopted in this study. Trait names in bold indicate which variables were selected over the others that correlated with it, whereas trait names in italic indicates which traits were removed from the final linear models. Here, "Acoust." is acousticness, "Dance" is danceability, "Instr." is instrumentalness, "Live." is liveliness, "Loud." is loudness, "Speech." is speechiness, and "BPM" is beats per minute.

	Duration	Key	Mode	*Acoust.*	Dance.	**Energy**	Instr.	Live.	*Loud.*	**Speech.**	Valence	BPM	Country	Pop	Pop/Rock	R&B	*Rap*	Rock	Trap
Duration	1.000	-0.094	-0.031	-0.220	-0.122	0.171	0.189	0.238	-0.048	0.130	-0.013	-0.297	-0.106	-0.102	-0.109	0.207	0.006	0.161	-0.193
Key	-0.094	1.000	-0.096	-0.170	0.240	0.308	0.029	0.233	0.284	0.170	0.327	0.026	-0.026	-0.049	-0.077	0.043	0.153	-0.232	-0.068
Mode	-0.031	-0.096	1.000	0.037	-0.306	-0.023	0.060	-0.054	-0.083	-0.048	-0.098	0.047	0.173	-0.063	0.098	-0.325	-0.146	0.208	-0.065
Acoust.	-0.220	-0.170	0.037	1.000	-0.048	**-0.726**	0.047	-0.183	-0.569	-0.063	-0.338	-0.238	-0.006	0.011	-0.163	-0.012	-0.064	-0.037	-0.044
Dance.	-0.122	0.240	-0.306	-0.048	1.000	-0.058	-0.295	0.185	0.078	0.567	0.235	-0.021	-0.272	-0.033	-0.217	0.086	0.598	-0.524	0.468
**Energy**	0.171	0.308	-0.023	**-0.726**	-0.058	1.000	-0.089	0.317	**0.770**	0.024	0.596	0.170	0.176	-0.036	0.258	-0.122	0.030	0.100	-0.158
Instr.	0.189	0.029	0.060	0.047	-0.295	-0.089	1.000	0.039	-0.278	-0.141	-0.127	0.066	-0.026	-0.154	-0.049	-0.071	-0.136	0.330	-0.069
Live.	0.238	0.233	-0.054	-0.183	0.185	0.317	0.039	1.000	0.211	0.566	0.196	-0.228	-0.181	-0.289	0.001	-0.008	0.498	-0.116	-0.029
Loud.	-0.048	0.284	-0.083	-0.569	0.078	**0.770**	-0.278	0.211	1.000	-0.045	0.405	0.222	0.105	0.173	0.162	-0.089	0.056	-0.209	0.010
**Speech.**	0.130	0.170	-0.048	-0.063	0.567	0.024	-0.141	0.566	-0.045	1.000	0.045	-0.194	-0.283	-0.365	-0.133	-0.065	**0.841**	-0.318	0.322
Valence	-0.013	0.327	-0.098	-0.338	0.235	0.596	-0.127	0.196	0.405	0.045	1.000	-0.088	0.201	-0.028	-0.036	0.026	-0.027	-0.068	-0.235
BPM	-0.297	0.026	0.047	-0.238	-0.021	0.170	0.066	-0.228	0.222	-0.194	-0.088	1.000	0.133	-0.010	0.144	-0.251	0.028	0.144	0.436
Country	-0.106	-0.026	0.173	-0.006	-0.272	0.176	-0.026	-0.181	0.105	-0.283	0.201	0.133	1.000	-0.372	-0.093	-0.149	-0.252	0.098	-0.127
Pop	-0.102	-0.049	-0.063	0.011	-0.033	-0.036	-0.154	-0.289	0.173	-0.365	-0.028	-0.010	-0.372	1.000	0.157	0.091	-0.442	-0.191	-0.220
Pop/Rock	-0.109	-0.077	0.098	-0.163	-0.217	0.258	-0.049	0.001	0.162	-0.133	-0.036	0.144	-0.093	0.157	1.000	-0.085	-0.143	0.474	-0.072
R&B	0.207	0.043	-0.325	-0.012	0.086	-0.122	-0.071	-0.008	-0.089	-0.065	0.026	-0.251	-0.149	0.091	-0.085	1.000	-0.230	-0.179	-0.116
Rap	0.006	0.153	-0.146	-0.064	0.598	0.030	-0.136	0.498	0.056	**0.841**	-0.027	0.028	-0.252	-0.442	-0.143	-0.230	1.000	-0.302	0.504
Rock	0.161	-0.232	0.208	-0.037	-0.524	0.100	0.330	-0.116	-0.209	-0.318	-0.068	0.144	0.098	-0.191	0.474	-0.179	-0.302	1.000	-0.152
Trap	-0.193	-0.068	-0.065	-0.044	0.468	-0.158	-0.069	-0.029	0.010	0.322	-0.235	0.436	-0.127	-0.220	-0.072	-0.116	0.504	-0.152	1.000

Table S3. Correlation matrix between trait at the level of albums. Correlation matrix between all traits at the album level (i.e., mean trait values considering all songs from each album of an artist). Values highlighted in bold represent correlation between two traits higher than the threshold of |0.6| adopted in this study. Trait names in bold indicate which variables were selected over the others that correlated with it, whereas trait names in italic indicates which traits were removed from the final linear models. Here, "Dur", is duration, "TimS." is time signature, "Acoust." is acousticness, "Dance" is danceability, "Instr." is instrumentalness, "Live." is liveliness, "Loud." is loudness, "Speech." is speechiness, "BPM" is beats per minute, and "P/R" is Pop/Rock.

	Dur.	Key	Mode	TimS.	*Acoust.*	Dance.	**Energy**	Instr.	Live.	*Loud.*	**Speech.**	Valence	BPM	Country	Pop	P/R	R&B	*Rap*	Rock	Trap
Dur.	1.000	0.005	-0.042	0.019	0.011	-0.239	0.004	0.336	0.119	-0.120	-0.014	-0.187	-0.139	-0.055	-0.099	-0.008	0.079	-0.030	0.182	-0.097
Key	0.005	1.000	-0.081	0.079	-0.074	0.007	0.109	-0.076	0.039	0.103	0.036	0.101	0.024	-0.028	0.034	-0.002	0.044	0.019	-0.082	-0.081
Mode	-0.042	-0.081	1.000	0.062	0.000	-0.190	0.036	-0.037	-0.011	0.002	-0.161	0.082	0.090	0.223	-0.075	0.104	-0.220	-0.226	0.256	-0.078
TimS.	0.019	0.079	0.062	1.000	-0.108	0.051	0.082	-0.230	-0.134	0.208	-0.118	0.025	0.031	0.021	0.039	0.013	0.019	-0.032	-0.012	0.015
Acoust.	0.011	-0.074	0.000	-0.108	1.000	-0.236	**-0.633**	0.272	-0.026	-0.517	-0.097	-0.213	-0.224	0.029	0.007	-0.116	-0.014	-0.097	0.032	-0.075
Dance.	-0.239	0.007	-0.190	0.051	-0.236	1.000	0.031	-0.286	-0.086	0.222	0.445	0.232	-0.020	-0.161	0.011	-0.146	0.070	0.508	-0.502	0.395
**Energy**	0.004	0.109	0.036	0.082	**-0.633**	0.031	1.000	-0.181	0.185	**0.750**	0.004	0.443	0.161	0.075	-0.003	0.146	-0.100	0.012	0.074	-0.128
Instr.	0.336	-0.076	-0.037	-0.230	0.272	-0.286	-0.181	1.000	0.135	-0.330	-0.082	-0.119	-0.077	-0.064	-0.074	-0.036	-0.053	-0.097	0.200	-0.041
Live.	0.119	0.039	-0.011	-0.134	-0.026	-0.086	0.185	0.135	1.000	-0.021	0.274	0.108	-0.067	-0.161	-0.143	-0.046	-0.080	0.220	0.019	-0.031
Loud.	-0.120	0.103	0.002	0.208	-0.517	0.222	**0.750**	-0.330	-0.021	1.000	-0.008	0.237	0.131	0.099	0.142	0.074	0.036	0.060	-0.211	0.022
**Speech.**	-0.014	0.036	-0.161	-0.118	-0.097	0.445	0.004	-0.082	0.274	-0.008	1.000	0.011	-0.171	-0.279	-0.297	-0.142	-0.074	**0.788**	-0.390	0.293
Valence	-0.187	0.101	0.082	0.025	-0.213	0.232	0.443	-0.119	0.108	0.237	0.011	1.000	-0.044	0.090	-0.007	-0.033	-0.054	-0.079	0.041	-0.227
BPM	-0.139	0.024	0.090	0.031	-0.224	-0.020	0.161	-0.077	-0.067	0.131	-0.171	-0.044	1.000	0.139	-0.045	0.121	-0.103	-0.024	0.126	0.262
Country	-0.055	-0.028	0.223	0.021	0.029	-0.161	0.075	-0.064	-0.161	0.099	-0.279	0.090	0.139	1.000	-0.275	-0.099	-0.131	-0.277	0.140	-0.129
Pop	-0.099	0.034	-0.075	0.039	0.007	0.011	-0.003	-0.074	-0.143	0.142	-0.297	-0.007	-0.045	-0.275	1.000	0.221	0.138	-0.376	-0.257	-0.184
P/R	-0.008	-0.002	0.104	0.013	-0.116	-0.146	0.146	-0.036	-0.046	0.074	-0.142	-0.033	0.121	-0.099	0.221	1.000	-0.095	-0.131	0.285	-0.077
R&B	0.079	0.044	-0.220	0.019	-0.014	0.070	-0.100	-0.053	-0.080	0.036	-0.074	-0.054	-0.103	-0.131	0.138	-0.095	1.000	-0.143	-0.230	-0.028
Rap	-0.030	0.019	-0.226	-0.032	-0.097	0.508	0.012	-0.097	0.220	0.060	**0.788**	-0.079	-0.024	-0.277	-0.376	-0.131	-0.143	1.000	-0.406	0.436
Rock	0.182	-0.082	0.256	-0.012	0.032	-0.502	0.074	0.200	0.019	-0.211	-0.390	0.041	0.126	0.140	-0.257	0.285	-0.230	-0.406	1.000	-0.195
Trap	-0.097	-0.081	-0.078	0.015	-0.075	0.395	-0.128	-0.041	-0.031	0.022	0.293	-0.227	0.262	-0.129	-0.184	-0.077	-0.028	0.436	-0.195	1.000

Table S4. Correlation matrix between trait at the level of tracks. Correlation matrix between all traits at the song level. Values highlighted in bold represent correlation between two traits higher than the threshold of |0.6| adopted in this study. Trait names in bold indicate which variables were selected over the others that correlated with it, whereas trait names in italic indicates which traits were removed from the final linear models. Here, “Dur”, is duration, “TimS.” is time signature, “Acoust.” is acousticness, “Dance” is danceability, “Instr.” is instrumentalness, “Live.” is liveliness, “Loud.” is loudness, “Speech.” is speechiness, “BPM” is beats per minute, and “P/R” is Pop/Rock.

	Dur.	Key	Mode	TimS.	*Acoust.*	Dance.	**Energy**	Instr.	Live.	*Loud.*	Speech.	Valence	BPM	Country	Pop	P/R	R&B	Rap	Rock	Trap
Dur.	1.000	-0.015	-0.028	0.105	-0.093	-0.109	0.050	0.014	-0.047	0.072	-0.129	-0.169	0.003	-0.023	-0.053	0.004	0.054	0.007	0.118	-0.053
Key	-0.015	1.000	-0.164	0.002	-0.021	0.008	0.039	-0.014	0.000	0.018	0.031	0.040	-0.019	-0.020	0.006	-0.003	-0.004	0.021	-0.017	-0.018
Mode	-0.028	-0.164	1.000	-0.026	0.065	-0.115	-0.048	0.026	-0.018	-0.043	-0.109	-0.016	0.023	0.193	-0.038	0.037	-0.127	-0.168	0.164	-0.080
TimS.	0.105	0.002	-0.026	1.000	-0.155	0.174	0.167	-0.057	-0.032	0.166	-0.013	0.100	0.039	0.000	-0.032	0.002	-0.008	0.056	-0.006	0.040
Acoust.	-0.093	-0.021	0.065	-0.155	1.000	-0.230	**-0.607**	0.112	-0.022	-0.500	0.015	-0.203	-0.151	0.033	0.020	-0.081	0.025	-0.103	-0.002	-0.078
Dance.	-0.109	0.008	-0.115	0.174	-0.230	1.000	0.100	-0.163	-0.056	0.185	0.231	0.328	-0.084	-0.118	-0.018	-0.102	0.034	0.365	-0.314	0.278
**Energy**	0.050	0.039	-0.048	0.167	**-0.607**	0.100	1.000	-0.082	0.131	**0.724**	0.002	0.419	0.156	0.045	-0.007	0.098	-0.098	0.050	0.041	-0.062
Instr.	0.014	-0.014	0.026	-0.057	0.112	-0.163	-0.082	1.000	0.056	-0.238	-0.085	-0.062	0.000	-0.062	-0.022	-0.018	-0.050	-0.112	0.175	-0.051
Live.	-0.047	0.000	-0.018	-0.032	-0.022	-0.056	0.131	0.056	1.000	-0.003	0.188	0.070	-0.009	-0.080	-0.079	-0.035	-0.042	0.124	-0.011	0.007
Loud.	0.072	0.018	-0.043	0.166	-0.500	0.185	**0.724**	-0.238	-0.003	1.000	-0.096	0.232	0.121	0.071	0.098	0.058	0.009	0.060	-0.133	0.039
Speech.	-0.129	0.031	-0.109	-0.013	0.015	0.231	0.002	-0.085	0.188	-0.096	1.000	0.087	-0.012	-0.224	-0.219	-0.127	-0.035	0.571	-0.307	0.207
Valence	-0.169	0.040	-0.016	0.100	-0.203	0.328	0.419	-0.062	0.070	0.232	0.087	1.000	0.038	0.056	0.011	0.004	-0.059	-0.033	0.022	-0.143
BPM	0.003	-0.019	0.023	0.039	-0.151	-0.084	0.156	0.000	-0.009	0.121	-0.012	0.038	1.000	0.045	-0.002	0.062	-0.051	-0.030	0.067	0.100
Country	-0.023	-0.020	0.193	0.000	0.033	-0.118	0.045	-0.062	-0.080	0.071	-0.224	0.056	0.045	1.000	-0.248	-0.079	-0.157	-0.293	0.140	-0.140
Pop	-0.053	0.006	-0.038	-0.032	0.020	-0.018	-0.007	-0.022	-0.079	0.098	-0.219	0.011	-0.002	-0.248	1.000	0.211	0.119	-0.376	-0.204	-0.188
P/R	0.004	-0.003	0.037	0.002	-0.081	-0.102	0.098	-0.018	-0.035	0.058	-0.127	0.004	0.062	-0.079	0.211	1.000	-0.086	-0.176	0.315	-0.090
R&B	0.054	-0.004	-0.127	-0.008	0.025	0.034	-0.098	-0.050	-0.042	0.009	-0.035	-0.059	-0.051	-0.157	0.119	-0.086	1.000	-0.038	-0.228	-0.003
Rap	0.007	0.021	-0.168	0.056	-0.103	0.365	0.050	-0.112	0.124	0.060	0.571	-0.033	-0.030	-0.293	-0.376	-0.176	-0.038	1.000	-0.407	0.421
Rock	0.118	-0.017	0.164	-0.006	-0.002	-0.314	0.041	0.175	-0.011	-0.133	-0.307	0.022	0.067	0.140	-0.204	0.315	-0.228	-0.407	1.000	-0.199
Trap	-0.053	-0.018	-0.080	0.040	-0.078	0.278	-0.062	-0.051	0.007	0.039	0.207	-0.143	0.100	-0.140	-0.188	-0.090	-0.003	0.421	-0.199	1.000

Table S5. Variable loadings from the Principal Component Analysis. Variable loadings of all PCA axis that summarized functional trait data. "P. of variance" is proportion of variance explained by each axis, and "Cumulative p." is the cumulative proportion of variance explained along the axis. Highlighted traits were the ones higher than |0.3| and thus selected to represent the direction of functional space in figure 2 of the main text.

Traits	PC1	PC2	PC3	PC4	PC5	PC6	PC7	PC8	PC9	PC10	PC11	PC12	PC13	PC14	PC15	PC16	PC17	PC18	PC19	PC20
**Acousticness**	**-0.316**	**-0.335**	**-0.022**	**0.187**	**-0.029**	**0.013**	**0.047**	**0.004**	**-0.025**	**-0.237**	**0.005**	**0.070**	**-0.082**	**0.073**	**0.211**	**-0.671**	**0.271**	**-0.248**	**-0.002**	**0.214**
Country	-0.138	0.187	0.329	0.238	-0.450	0.150	-0.030	-0.225	-0.016	-0.034	-0.013	-0.135	-0.179	0.012	0.549	0.171	-0.190	-0.026	0.291	-0.007
**Danceability**	**0.373**	**-0.073**	**-0.084**	**0.214**	**-0.146**	**-0.143**	**0.307**	**0.214**	**0.116**	**0.143**	**-0.054**	**-0.047**	**0.067**	**0.405**	**0.142**	**0.303**	**0.306**	**-0.414**	**-0.068**	**0.175**
Duration	-0.037	0.083	0.007	-0.582	-0.147	0.288	-0.028	0.261	-0.177	-0.002	-0.001	0.380	0.126	0.450	0.244	-0.077	-0.128	0.027	-0.051	0.007
**Energy**	**0.315**	**0.441**	**0.072**	**-0.016**	**0.130**	**0.109**	**-0.156**	**-0.006**	**-0.008**	**0.110**	**0.040**	**0.025**	**0.000**	**-0.182**	**-0.003**	**-0.209**	**-0.045**	**-0.053**	**0.067**	**0.740**
Instrumentalness	-0.181	-0.046	0.116	-0.089	0.325	0.129	-0.014	0.234	0.643	0.364	0.222	0.009	0.065	-0.182	0.351	0.007	0.037	-0.049	-0.009	-0.087
Key	0.045	0.002	-0.080	0.018	0.170	0.451	0.437	-0.505	-0.035	-0.099	0.296	-0.095	0.448	0.057	0.011	0.008	0.021	-0.005	-0.003	-0.001
Liveliness	0.085	-0.039	0.178	0.079	0.453	0.212	-0.452	0.120	-0.123	-0.311	0.126	-0.422	-0.096	0.373	0.068	0.123	0.092	-0.017	-0.013	-0.049
**Loudness**	**0.320**	**0.388**	**-0.088**	**-0.072**	**-0.122**	**0.015**	**-0.176**	**-0.092**	**-0.102**	**0.120**	**0.154**	**-0.070**	**-0.040**	**-0.141**	**0.088**	**-0.348**	**0.225**	**-0.349**	**-0.121**	**-0.537**
Mode	-0.163	0.093	0.248	0.168	-0.190	-0.349	-0.224	0.213	-0.109	-0.049	0.308	-0.003	0.713	-0.010	-0.032	-0.013	0.030	0.033	0.003	0.019
Pop	-0.087	0.127	-0.610	0.101	0.143	-0.223	-0.075	0.010	0.025	-0.074	0.347	0.125	-0.075	0.168	0.069	0.027	-0.275	-0.124	0.495	-0.019
Pop-Rock	-0.103	0.220	-0.095	-0.142	0.367	-0.403	0.296	0.009	-0.334	-0.042	-0.203	-0.167	0.048	-0.116	0.516	0.028	0.104	0.180	-0.135	0.008
R&B	0.023	-0.095	-0.427	-0.105	-0.164	0.197	-0.257	0.057	0.165	-0.076	-0.536	-0.348	0.401	-0.154	0.121	-0.016	-0.032	-0.060	0.129	0.026
**Rap**	**0.393**	**-0.318**	**0.181**	**-0.151**	**0.055**	**-0.029**	**0.013**	**0.029**	**-0.126**	**0.051**	**0.013**	**0.115**	**0.053**	**-0.141**	**0.081**	**-0.055**	**0.349**	**0.251**	**0.654**	**-0.085**
Rock	-0.288	0.254	0.321	-0.138	0.204	-0.011	0.259	0.097	-0.030	0.049	-0.300	-0.134	0.061	0.086	-0.332	-0.067	-0.073	-0.437	0.412	-0.101
**Speechiness**	**0.292**	**-0.308**	**0.162**	**0.106**	**0.236**	**-0.024**	**-0.047**	**0.010**	**-0.145**	**-0.176**	**-0.107**	**0.310**	**0.121**	**-0.272**	**0.182**	**0.064**	**-0.501**	**-0.412**	**-0.103**	**-0.080**
Tempo	0.047	0.148	0.124	-0.212	0.060	-0.316	-0.179	-0.464	0.465	-0.407	-0.169	0.285	0.059	0.159	0.026	0.084	0.188	-0.044	-0.009	0.007
Time Signature	0.151	0.125	0.016	-0.154	-0.182	0.041	0.337	0.416	0.220	-0.641	0.211	-0.173	-0.105	-0.257	-0.035	-0.011	-0.053	0.043	0.007	-0.018
Trap	0.242	-0.231	0.127	-0.262	-0.098	-0.337	0.087	-0.169	0.159	0.171	0.104	-0.448	-0.010	0.262	0.001	-0.344	-0.428	0.073	-0.028	0.036
Valence	0.212	0.240	0.037	0.493	0.132	0.084	0.137	0.165	0.189	-0.031	-0.298	0.185	0.108	0.271	0.011	-0.323	-0.173	0.391	0.022	-0.224
P. of variance	*0.144*	*0.126*	*0.081*	*0.066*	*0.065*	*0.057*	*0.054*	*0.052*	*0.048*	*0.043*	*0.041*	*0.039*	*0.038*	*0.033*	*0.029*	*0.026*	*0.021*	*0.018*	*0.010*	*0.009*
Cumulative p.	*0.144*	*0.270*	*0.351*	*0.417*	*0.482*	*0.539*	*0.593*	*0.645*	*0.693*	*0.737*	*0.778*	*0.816*	*0.854*	*0.887*	*0.917*	*0.942*	*0.963*	*0.981*	*0.991*	*1.000*

Table S6. Selection steps for functional diversity models. Steps of model selection using both backward and forward selection. Full model is log10(popularity) ~ Richness * Evenness * Divergence + Year of career launch + Richness:Year of career launch + Evenness:Year of career launch + Divergence:Year of career launch. “DF” is degrees of freedom, “Residuals DF” is residuals of the degrees of freedom, and “AIC” is Akaike Information Criteria. Bold line indicates the selected model.

	Step	DF	Deviance	Residuals DF	Residuals Deviance	AIC
1				88	8.56227	-221.78043
2	- Divergence:*Year of career launch*	1	0.01644	89	8.57872	-223.58860
3	- Evenness:*Year of career launch*	1	0.08827	90	8.66698	-224.56493
**4**	**- Richness:***Year of career launch*	**1**	**0.08747**	**91**	**8.75446**	**-225.56070**

Table S7. Coefficients of models in which functional diversity is the predictor. Full coefficients of the selected model, which has a structure of log10(popularity) ~ Richness + Evenness + Divergence + Year of career launch + Richness:Evenness + Richness:Divergence + Evenness:Divergence + Richness:Evenness:Divergence. “Std. Error” is Standard Error. Adjusted R-squared and P value of the full model is provided in the main text. Bold lines represent traits that had a significant effect on the total number of streams assuming a significance level of 0.05.

	Estimate	Std. Error	t value	P
(Intercept)	9.72300	0.03449	281.91699	1.19E-135
**Richness**	**0.13058**	**0.03685**	**3.54297**	**0.00063**
Evenness	-0.01698	0.03527	-0.48145	0.63136
Divergence	-0.02367	0.04152	-0.57011	0.57001
**Year of career launch**	**0.08640**	**0.03183**	**2.71400**	**0.00795**
Richness:Evenness	0.03834	0.03927	0.97618	0.33156
Richness:Divergence	0.00497	0.03811	0.13042	0.89652
Evenness:Divergence	-0.00286	0.03607	-0.07933	0.93695
Richness:Evenness:Divergence	-0.04027	0.02620	-1.53674	0.12783

Table S8. Selection steps for models in which traits are the predictors. Steps of model selection using both backward and forward selection at three levels of organization, that is, artists, albums and tracks. Full model is log10(popularity) ~ Duration + Key + Mode + Danceability + Energy + Instrumentalness + Liveliness + Speechiness + Valence + Beats per minutes + Country + Pop + Pop/Rock + R&B + Rock + Trap. “Df” is degrees of freedom, “Resid. Df” is residuals of the degrees of freedom, “Resid. Dev.” is the residuals of deviance, and “AIC” is Akaike Information Criteria. Bold line indicates the selected model.

Step		Df	Deviance	Resid. Df	Resid. Dev	AIC
			Artists			
1				83	5.69451	-252.56683
2	- R&B	1	0.01031	84	5.70481	-254.38602
3	- Trap	1	0.01280	85	5.71761	-256.16188
4	- Duration	1	0.02762	86	5.74524	-257.67989
5	- Energy	1	0.03822	87	5.78346	-259.01680
6	- Pop	1	0.06996	88	5.85342	-259.81446
**7**	**- Key**	**1**	**0.11655**	**89**	**5.96997**	**-259.84283**
			Albums			
1				770	477.27274	-359.61030
2	- Duration	1	0.12689	771	477.39963	-361.40108
3	- Key	1	0.29784	772	477.69747	-362.91024
4	- Mode	1	0.52930	773	478.22677	-364.03871
5	- Country	1	0.54387	774	478.77064	-365.14419
**6**	**- Tempo**	**1**	**1.05259**	**775**	**479.82323**	**-365.41585**
			Tracks			
1				10335	8937.26417	-1486.37564
**2**	**- Key**	**1**	**1.08847**	**10336**	**8938.35264**	**-1487.11482**

Table S9. Coefficients of models in which traits are the predictors. Full coefficients of the selected models at the three levels of organization, that is, artists, albums and tracks. "Std. Error" is Standard Error. Adjusted R-squared and P value of the full model is provided in the main text. Bold lines represent traits that had a significant effect on the total number of streams assuming a significance level of 0.05.

	Estimate	Std. Error	t value	P
Artists				
(Intercept)	9.64514	0.07341	131.38101	1.06E-103
**Mode**	**0.19116**	**0.08020**	**2.38362**	**0.01927**
**Danceability**	**0.12530**	**0.04135**	**3.03054**	**0.00320**
Instrumentalness	0.04731	0.02954	1.60165	0.11278
**Liveliness**	**0.07670**	**0.03452**	**2.22213**	**0.02881**
**Speechiness**	**-0.12816**	**0.04098**	**-3.12753**	**0.00238**
**Valence**	**-0.15003**	**0.02980**	**-5.03428**	**2.48E-06**
Tempo	-0.04390	0.02797	-1.56964	0.12005
**Country**	**-0.28524**	**0.08601**	**-3.31637**	**0.00132**
**Pop/Rock**	**0.41792**	**0.14379**	**2.90647**	**0.00461**
**Rock**	**-0.25720**	**0.09139**	**-2.81443**	**0.00601**
Albums				
(Intercept)	8.24716	0.05529	149.16057	0.00000
**Danceability**	**0.15190**	**0.04093**	**3.71111**	**0.00022**
**Instrumentalness**	**-0.10527**	**0.03040**	**-3.46334**	**0.00056**
**Liveliness**	**-0.07544**	**0.03125**	**-2.41399**	**0.01601**
**Energy**	**0.16480**	**0.03293**	**5.00374**	**6.96E-07**
**Speechiness**	**-0.14303**	**0.03837**	**-3.72794**	**0.00021**
**Valence.**	**-0.30540**	**0.03515**	**-8.68887**	**2.16E-17**
**Pop**	**0.40420**	**0.07235**	**5.58666**	**3.21E-08**
Pop/Rock	0.20539	0.13602	1.50998	0.13145
**R&B**	**0.23948**	**0.09158**	**2.61486**	**0.00910**
**Rock**	**-0.25263**	**0.08927**	**-2.82976**	**0.00478**
Trap	0.17793	0.12367	1.43876	0.15062
Tracks				
(Intercept)	5.49669	0.10273	53.50782	0.00000
**Duration**	**0.07626**	**0.00960**	**7.94531**	**2.14E-15**
**Mode**	**-0.06165**	**0.02003**	**-3.07767**	**0.00209**
**Time signature**	**0.28447**	**0.02538**	**11.20876**	**5.43E-29**
**Danceability**	**0.08524**	**0.01121**	**7.60638**	**3.06E-14**
**Energy**	**0.08072**	**0.01059**	**7.62247**	**2.71E-14**

**Instrumentalness**	**-0.07547**	**0.00948**	**-7.96213**	**1.87E-15**
**Liveliness**	**-0.04398**	**0.00954**	**-4.61141**	**4.05E-06**
**Speechiness**	**-0.06309**	**0.01091**	**-5.78171**	**7.61E-09**
**Valence**	**-0.15879**	**0.01129**	**-14.05982**	**1.72E-44**
Tempo	0.01540	0.00947	1.62635	0.10390
**Country**	**0.23838**	**0.02958**	**8.05770**	**8.63E-16**
**Pop**	**0.54447**	**0.02271**	**23.97771**	**1.09E-123**
**Pop/Rock**	**0.45636**	**0.04089**	**11.16012**	**9.33E-29**
**R&B**	**0.40345**	**0.02698**	**14.95496**	**4.82E-50**
**Rock**	**-0.06168**	**0.02708**	**-2.27781**	**0.02276**
**Trap**	**0.66000**	**0.03399**	**19.41842**	**1.58E-82**

Table S10. Selection steps for models in which traits are the predictors per musical genre. Steps of model selection using both backward and forward selection at the three levels of organization (i.e., artists, albums and tracks) separated for the seven major musical genres in our dataset (i.e., Country, Pop, Pop/Rock, Rock, R&B, Trap and Rap). Global model is log10(popularity of artists, albums or tracks per musical genre) ~ Duration + Key + Mode + Danceability + Energy + Instrumentalness + Liveliness + Speechiness + Valence + Beats per minutes. "Df" is degrees of freedom, "Resid. Df" is residuals of the degrees of freedom, "Resid. Dev." is the residuals of deviance, and "AIC" is Akaike Information Criteria. Bold line indicates the selected model.

Step	Df	Deviance	Resid. Df	Resid. Dev.	AIC	Musical genre
			Artist			
			3	0.060364843	-54.2496805	Country
- Mode	0	0	3	0.060364843	-54.2496805	Country
- Key	1	3.12E-05	4	0.060396037	-56.24244759	Country
- Energy	1	0.000495661	5	0.060891698	-58.12802061	Country
- Liveliness	1	0.009022737	6	0.069914435	-58.19356653	Country
**- Valence**	**1**	**0.00549123**	**7**	**0.075405666**	**-59.13502272**	**Country**
			34	2.288189339	-114.0405064	Pop
- Duration	1	0.001823303	35	2.290012642	-116.0038667	Pop
- Instrumentalness	1	0.023113338	36	2.313125979	-117.5419113	Pop
- Key	1	0.037033092	37	2.350159072	-118.8112855	Pop
**- Tempo**	**1**	**0.047141486**	**38**	**2.397300558**	**-119.8977107**	**Pop**
			16	1.061077833	-67.64174027	Rap
- Key	1	0.000605444	17	1.061683277	-69.6257682	Rap
- Instrumentalness	1	0.002774362	18	1.064457639	-71.55269479	Rap
- Danceability	1	0.013117411	19	1.07757505	-73.20975689	Rap
- Acousticness	1	0.009241242	20	1.086816292	-74.9706538	Rap
**- Duration**	**1**	**0.05458694**	**21**	**1.141403232**	**-75.59849079**	**Rap**
			8	0.374054905	-52.62804165	Rock
- Mode	0	0	8	0.374054905	-52.62804165	Rock
- Acousticness	1	2.46E-05	9	0.37407948	-54.62679341	Rock
- Tempo	1	0.000501326	10	0.374580806	-56.60134745	Rock
- Liveliness	1	0.002337873	11	0.376918679	-58.48313119	Rock
- Danceability	1	0.006288609	12	0.383207288	-60.1687457	Rock
- Key	1	0.009491248	13	0.392698535	-61.70388851	Rock
**- Speechiness**	**1**	**0.01968537**	**14**	**0.412383905**	**-62.77455115**	**Rock**
			Album			
			107	26.50453346	-154.7151164	Country

- Duration	1	0.002920961	108	26.50745443	-156.7020026	Country
- Liveliness	1	0.013095635	109	26.52055006	-158.6432268	Country
- Tempo	1	0.089068188	110	26.60961825	-160.2442398	Country
- Key	1	0.162821362	111	26.77243961	-161.5183104	Country
- Mode	1	0.20703825	112	26.97947786	-162.6015924	Country
- Energy	1	0.281171411	113	27.26064927	-163.3678307	Country
- Valence	1	0.12423984	114	27.38488911	-164.8267228	Country
- Danceability	1	0.284477479	115	27.66936659	-165.5969133	Country
**- Instrumentalness**	**1**	**0.338659795**	**116**	**28.00802639**	**-166.1492517**	**Country**
			286	137.1976083	-207.1500187	Pop
- Liveliness	1	0.016128292	287	137.2137366	-209.1149893	Pop
- Key	1	0.04957322	288	137.2633098	-211.007346	Pop
- Instrumentalness	1	0.05804856	289	137.3213583	-212.8813486	Pop
- Speechiness	1	0.095543509	290	137.4169019	-214.6740825	Pop
- Acousticness	1	0.153849987	291	137.5707518	-216.3406326	Pop
- Duration	1	0.46494054	292	138.0356924	-217.3351959	Pop
**- Mode**	**1**	**0.84984115**	**293**	**138.8855335**	**-217.5061309**	**Pop**
			33	13.88273479	-28.92074355	Pop/Rock
- Key	1	0.026730165	34	13.90946495	-30.83418273	Pop/Rock
- Mode	1	0.057173123	35	13.96663807	-32.64959494	Pop/Rock
- Danceability	1	0.061020995	36	14.02765907	-34.45341576	Pop/Rock
- Instrumentalness	1	0.080906392	37	14.10856546	-36.19461788	Pop/Rock
- Acousticness	1	0.097129949	38	14.20569541	-37.88587842	Pop/Rock
- Duration	1	0.339989524	39	14.54568493	-38.82156487	Pop/Rock
**- Liveliness**	**1**	**0.423207906**	**40**	**14.96889284**	**-39.53097137**	**Pop/Rock**
			79	33.43315996	-67.11932701	R&B
- Duration	1	0.01430833	80	33.44746829	-69.08039023	R&B
- Energy	1	0.041206544	81	33.48867484	-70.96834924	R&B
- Key	1	0.098582985	82	33.58725782	-72.70085961	R&B
- Tempo	1	0.268164467	83	33.85542229	-73.97719042	R&B
- Instrumentalness	1	0.701409448	84	34.55683174	-74.11113594	R&B
**- Acousticness**	**1**	**0.409486945**	**85**	**34.96631868**	**-75.03915509**	**R&B**
			224	208.8337624	-4.861210527	Rap
- Instrumentalness	1	0.029430714	225	208.8631931	-6.827953648	Rap
- Energy	1	0.049491115	226	208.9126842	-8.772038963	Rap
- Key	1	0.151062362	227	209.0637466	-10.60145174	Rap
- Mode	1	0.37224443	228	209.435991	-12.18162013	Rap
- Acousticness	1	0.49494013	229	209.9309311	-13.62456177	Rap
**- Danceability**	**1**	**0.563761036**	**230**	**210.4946922**	**-14.99164277**	**Rap**
			212	117.9749516	-119.6229142	Rock

- Liveliness	1	0.016464447	213	117.9914161	-121.5916552	Rock
- Mode	1	0.044061427	214	118.0354775	-123.5080227	Rock
- Acousticness	1	0.296656388	215	118.3321339	-124.9457539	Rock
- Key	1	0.341351837	216	118.6734857	-126.300513	Rock
**- Duration**	**1**	**0.625340527**	**217**	**119.2988263**	**-127.1232617**	**Rock**
			50	28.82480337	-23.48568471	Trap
- Key	1	0.000328734	51	28.8251321	-25.48497763	Trap
- Instrumentalness	1	0.014887858	52	28.84001996	-27.45296359	Trap
- Mode	1	0.0425137	53	28.88253366	-29.36163534	Trap
- Acousticness	1	0.14092429	54	29.02345795	-31.0598592	Trap
- Danceability	1	0.090374925	55	29.11383288	-32.86709997	Trap
- Duration	1	0.28899466	56	29.40282754	-34.25469952	Trap
- Valence	1	0.482392399	57	29.88521994	-35.24576129	Trap
**- Tempo**	**1**	**0.730828926**	**58**	**30.61604886**	**-35.74782254**	**Trap**
			Track			
			1503	945.9875358	-689.4761068	Country
- Tempo	1	0.005995576	1504	945.9935314	-691.4665049	Country
- Acousticness	1	0.010899013	1505	946.0044304	-693.4490503	Country
- Key	1	0.179770327	1506	946.1842008	-695.1611805	Country
- Speechiness	1	0.213010909	1507	946.3972117	-696.8201526	Country
**- Valence**	**1**	**0.689221045**	**1508**	**947.0864327**	**-697.7172437**	**Country**
			4095	3857.267662	-233.6475595	Pop
**- Tempo**	**1**	**0.106635935**	**4096**	**3857.374298**	**-235.5340211**	**Pop**
			686	480.7420552	-236.2759945	Pop/Rock
- Speechiness	1	0.00156343	687	480.7436186	-238.2737245	Pop/Rock
- Acousticness	1	0.015771973	688	480.7593906	-240.2508253	Pop/Rock
- Instrumentalness	1	0.016888414	689	480.776279	-242.2263059	Pop/Rock
- Key	1	0.11457419	690	480.8908532	-244.0599848	Pop/Rock
- Liveliness	1	0.203986547	691	481.0948398	-245.7639666	Pop/Rock
**- Tempo**	**1**	**0.627070344**	**692**	**481.7219101**	**-246.8547694**	**Pop/Rock**
			1560	1136.76812	-485.5786402	R&B
- Instrumentalness	1	0.150193198	1561	1136.918313	-487.3709566	R&B
- Key	1	0.493023175	1562	1137.411336	-488.6894086	R&B
- Energy	1	0.529559509	1563	1137.940896	-489.9576823	R&B
- Acousticness	1	0.902187739	1564	1138.843083	-490.7118555	R&B
**- Duration**	**1**	**0.845434775**	**1565**	**1139.688518**	**-491.5452941**	**R&B**
			3538	4246.334862	659.835608	Rap
- Key	1	0.11031278	3539	4246.445175	657.9278299	Rap
- Energy	1	0.259907149	3540	4246.705082	656.1451039	Rap
**- Instrumentalness**	**1**	**0.604185887**	**3541**	**4247.309268**	**654.6501325**	**Rap**

			2630	2098.47079	-584.5247843	Rock
- Acousticness	1	0.179543773	2631	2098.650334	-586.2987462	Rock
- Key	1	0.302187177	2632	2098.952521	-587.9183488	Rock
**- Speechiness**	**1**	**0.359655256**	**2633**	**2099.312176**	**-589.4656812**	**Rock**
			1079	745.7090267	-391.1413265	Trap
- Key	1	0.002287025	1080	745.7113137	-393.1379805	Trap
- Danceability	1	0.006821194	1081	745.7181349	-395.1280009	Trap
- Valence	1	0.178267394	1082	745.8964023	-396.8672234	Trap
- Acousticness	1	0.277578597	1083	746.1739809	-398.4612932	Trap
- Mode	1	0.491261566	1084	746.6652425	-399.7432435	Trap
- Tempo	1	0.65602026	1085	747.3212627	-400.7851114	Trap
**- Instrumentalness**	**1**	**1.204576507**	**1086**	**748.5258392**	**-401.027989**	**Trap**

Table S11. Coefficients of models in which traits are the predictors separated by the seven major musical genres. Full coefficients of the selected models at each of the three levels of organization (i.e., artists, albums and tracks) for each of the seven major musical genres in our dataset (i.e., Country, Pop, Pop/Rock, Rock, R&B, Rap, and Trap), which has a structure of log10(popularity of artists, albums or tracks per musical genre) ~ Danceability + Energy + Instrumentalness + Liveliness + Speechiness + Valence. "Std. Error" is Standard Error. Adjusted R-squared and P value of the full model is provided in the main text. Bold lines represent traits that had a significant effect on the total number of streams assuming a significance level of 0.05.

Variable	Estimate	Std. Error	T	P	Musical genre
			Artist		
(Intercept)	10.610832	0.450411384	23.55809016	6.31E-08	Country
**Duration**	**-0.14193694**	**0.055747643**	**-2.546061733**	**0.038323301**	**Country**
Acousticness	-0.120055883	0.088815945	-1.351737951	0.218507802	Country
Danceability	0.156181793	0.073288409	2.131057204	0.070559959	Country
**Instrumentalness**	**0.181651611**	**0.073601409**	**2.468045292**	**0.04295584**	**Country**
**Speechiness**	**1.728833152**	**0.626925484**	**2.757637383**	**0.028191818**	**Country**
**Tempo**	**0.090437164**	**0.038367304**	**2.35714149**	**0.050553446**	**Country**
(Intercept)	9.600929586	0.095206459	100.8432591	9.05E-48	Pop
Mode	0.158995283	0.111880239	1.421120334	0.163436653	Pop
Acousticness	0.091953618	0.061925604	1.484904651	0.1458181	Pop
**Danceability**	**0.269034135**	**0.070454849**	**3.818532555**	**0.0004819**	**Pop**
Energy	0.111785194	0.076289856	1.46526943	0.151073209	Pop
**Liveliness**	**0.158987243**	**0.075621866**	**2.102397786**	**0.04219898**	**Pop**
**Speechiness**	**-0.43443475**	**0.115722262**	**-3.754115603**	**0.000581392**	**Pop**
**Valence**	**-0.24156426**	**0.052850848**	**-4.570678999**	**5.03E-05**	**Pop**
(Intercept)	9.816892687	0.101748522	96.48191903	2.73E-29	Rap
Mode	0.269668617	0.142244708	1.895807726	0.071831513	Rap
**Energy**	**-0.180204362**	**0.085185632**	**-2.115431409**	**0.046514639**	**Rap**
Liveliness	0.07054643	0.044509078	1.584989691	0.127913053	**Rap**
**Speechiness**	**-0.19594734**	**0.070016424**	**-2.798591086**	**0.010763601**	**Rap**
Valence	-0.140042289	0.071423881	-1.960720814	0.063310179	Rap
**Tempo**	**-0.112929624**	**0.042700648**	**-2.644681753**	**0.015153572**	**Rap**
(Intercept)	9.532053718	0.0442474	215.4263029	4.75E-26	Rock
**Duration**	**-0.179894311**	**0.050748487**	**-3.544821194**	**0.003234019**	**Rock**
**Energy**	**0.213712633**	**0.042376823**	**5.043149022**	**0.000179578**	**Rock**
**Instrumentalness**	**0.071927495**	**0.020320756**	**3.539607269**	**0.003267683**	**Rock**
**Valence**	**-0.246113029**	**0.046490819**	**-5.293798573**	**0.00011344**	**Rock**

		Album			
(Intercept)	9.168861632	0.34005221	26.9630997	8.72E-52	Country
**Acousticness**	**-0.1920072**	**0.049316551**	**-3.893362261**	**0.000165612**	**Country**
**Speechiness**	**1.354352519**	**0.51051288**	**2.652925266**	**0.009097816**	**Country**
(Intercept)	8.761450649	0.040007828	218.993408	0	Pop
**Danceability**	**0.289670676**	**0.06291871**	**4.603887711**	**6.19E-06**	**Pop**
**Energy**	**0.117638259**	**0.050458352**	**2.331393192**	**0.02041066**	**Pop**
**Valence**	**-0.449737798**	**0.05178827**	**-8.684163393**	**2.72E-16**	**Pop**
Tempo	-0.082655649	0.053661706	-1.540309753	0.124564124	Pop
(Intercept)	7.878034574	0.506538642	15.55268232	1.48E-18	Pop/Rock
**Energy**	**0.265313888**	**0.128487309**	**2.064903448**	**0.045456107**	**Pop/Rock**
Speechiness	-1.298763712	0.789696707	-1.6446361	0.107882448	Pop/Rock
**Valence**	**-0.27215054**	**0.106208039**	**-2.562428808**	**0.014264339**	**Pop/Rock**
Tempo	-0.148203391	0.103789228	-1.427926511	0.161074554	Pop/Rock
(Intercept)	8.454271676	0.126040341	67.07591852	2.17E-75	R&B
**Mode**	**0.3107421**	**0.147046566**	**2.113222412**	**0.037513715**	**R&B**
Danceability	0.172646785	0.125331666	1.377519268	0.171968121	R&B
Liveliness	-0.202967655	0.117089115	-1.733445969	0.086642503	R&B
Speechiness	-0.280058558	0.157165837	-1.78193024	0.078331897	R&B
**Valence**	**-0.24553538**	**0.091942248**	**-2.670539231**	**0.009072782**	**R&B**
(Intercept)	8.540921661	0.100825701	84.70976699	2.12E-175	Rap
**Duration**	**0.215987247**	**0.083011675**	**2.601889999**	**0.009873054**	**Rap**
**Liveliness**	**-0.163677173**	**0.062564987**	**-2.616114569**	**0.009482621**	**Rap**
**Speechiness**	**-0.184507727**	**0.065366328**	**-2.822672368**	**0.005179647**	**Rap**
**Valence**	**-0.373931794**	**0.072764275**	**-5.138947595**	**5.90E-07**	**Rap**
Tempo	-0.085208089	0.059595182	-1.429781506	0.154136679	Rap
(Intercept)	7.82791131	0.234625535	33.36342442	2.16E-87	Rock
**Danceability**	**0.245122818**	**0.07235895**	**3.387594996**	**0.000837142**	**Rock**
**Energy**	**0.252388233**	**0.05039467**	**5.008232711**	**1.14E-06**	**Rock**
Instrumentalness	-0.064142934	0.034163965	-1.877502661	0.061790766	Rock
Speechiness	-0.62600073	0.346772857	-1.805218365	0.072426597	Rock
**Valence**	**-0.307330011**	**0.054280131**	**-5.661924613**	**4.71E-08**	**Rock**
**Tempo**	**0.142450485**	**0.055310855**	**2.575452584**	**0.010675184**	**Rock**
(Intercept)	8.728069325	0.136971262	63.72190202	2.15E-55	Trap
**Energy**	**-0.4485732**	**0.18353012**	**-2.444139417**	**0.017581367**	**Trap**
**Liveliness**	**-0.359297289**	**0.136157308**	**-2.638839547**	**0.010665263**	**Trap**
**Speechiness**	**-0.265109775**	**0.106595021**	**-2.487074657**	**0.01577836**	**Trap**
		Track			
(Intercept)	6.985097671	0.062203228	112.2947781	0	Country
**Duration**	**0.129891796**	**0.030515167**	**4.256630628**	**2.20E-05**	**Country**

**Mode**	**-0.167168619**	**0.064397228**	**-2.595897742**	**0.00952604**	**Country**
**Danceability**	**0.063059618**	**0.029043257**	**2.171230932**	**0.030069222**	**Country**
**Energy**	**0.126417735**	**0.020606345**	**6.134893611**	**1.09E-09**	**Country**
**Instrumentalness**	**-0.149839439**	**0.039380484**	**-3.804916135**	**0.000147523**	**Country**
**Liveliness**	**-0.064752858**	**0.026203039**	**-2.471196476**	**0.01357599**	**Country**
(Intercept)	7.401593801	0.035946209	205.9074954	0	Pop
**Duration**	**0.102491501**	**0.017654817**	**5.805299709**	**6.91E-09**	**Pop**
Key	-0.00678043	0.004259468	-1.59184914	0.111495771	Pop
**Mode**	**-0.121994047**	**0.032133027**	**-3.796531459**	**0.000148858**	**Pop**
**Acousticness**	**0.041778557**	**0.020736188**	**2.014765536**	**0.043994448**	**Pop**
**Danceability**	**0.173739415**	**0.019010789**	**9.138990005**	**9.69E-20**	**Pop**
**Energy**	**0.094780022**	**0.022563909**	**4.20051418**	**2.72E-05**	**Pop**
**Instrumentalness**	**-0.165204551**	**0.016548351**	**-9.983142724**	**3.32E-23**	**Pop**
**Liveliness**	**-0.039269872**	**0.016623891**	**-2.362255107**	**0.018210647**	**Pop**
**Speechiness**	**-0.052292051**	**0.023933479**	**-2.184891397**	**0.028953292**	**Pop**
**Valence**	**-0.226665908**	**0.019171102**	**-11.82331159**	**9.65E-32**	**Pop**
(Intercept)	7.614784613	0.064029553	118.9260936	0	Pop/Rock
**Duration**	**-0.12565973**	**0.046670653**	**-2.692478479**	**0.007263895**	**Pop/Rock**
Mode	-0.114733661	0.071711517	-1.599933536	0.11006975	Pop/Rock
**Danceability**	**0.250834826**	**0.043677998**	**5.742818787**	**1.39E-08**	**Pop/Rock**
**Energy**	**0.118941533**	**0.03637358**	**3.269998012**	**0.001128762**	**Pop/Rock**
**Valence**	**-0.222705996**	**0.040989483**	**-5.433247283**	**7.67E-08**	**Pop/Rock**
(Intercept)	7.324080584	0.031584899	231.8855153	0	R&B
Mode	0.063295294	0.043223207	1.464382185	0.143290391	R&B
**Danceability**	**0.139991427**	**0.026311655**	**5.320510054**	**1.18E-07**	**R&B**
Liveliness	-0.047747942	0.024773669	-1.927366584	0.054114774	R&B
Speechiness	-0.044275073	0.031000029	-1.42822682	0.153426112	R&B
**Valence**	**-0.150520775**	**0.025596257**	**-5.880577581**	**4.99E-09**	**R&B**
**Tempo**	**0.054314881**	**0.022088559**	**2.458959923**	**0.014041595**	**R&B**
(Intercept)	7.023526661	0.032049604	219.1455046	0	Rap
**Duration**	**0.172031495**	**0.018429904**	**9.334367313**	**1.74E-20**	**Rap**
Mode	-0.069328057	0.037145032	-1.866415312	0.062065881	Rap
Acousticness	0.034281616	0.023692582	1.446934556	0.148003769	Rap
**Danceability**	**0.130136929**	**0.020949892**	**6.211818601**	**5.84E-10**	**Rap**
**Liveliness**	**-0.046676276**	**0.017375705**	**-2.6862954**	**0.007258651**	**Rap**
**Speechiness**	**-0.131765612**	**0.016040873**	**-8.214366819**	**2.96E-16**	**Rap**
**Valence**	**-0.207258575**	**0.019817553**	**-10.45833338**	**3.12E-25**	**Rap**
**Tempo**	**0.082491363**	**0.018158492**	**4.542853164**	**5.73E-06**	**Rap**
(Intercept)	6.939210084	0.039909892	173.8719325	0	Rock
**Duration**	**-0.048430136**	**0.016170372**	**-2.994992091**	**0.002770093**	**Rock**

Mode	-0.06985373	0.042946528	-1.626527979	0.103957049	Rock
**Danceability**	**0.183454074**	**0.024126018**	**7.603992999**	**3.97E-14**	**Rock**
**Energy**	**0.201101333**	**0.017761467**	**11.32233805**	**4.72E-29**	**Rock**
**Instrumentalness**	**-0.072416229**	**0.011738452**	**-6.169146427**	**7.92E-10**	**Rock**
**Liveliness**	**-0.05656595**	**0.017839487**	**-3.170828294**	**0.001537521**	**Rock**
**Valence**	**-0.25378873**	**0.021758509**	**-11.66388451**	**1.09E-30**	**Rock**
**Tempo**	**0.060548241**	**0.0190031**	**3.186229749**	**0.001458267**	**Rock**
(Intercept)	7.516589745	0.029852146	251.7939522	0	Trap
**Duration**	**0.080831973**	**0.031654325**	**2.553583852**	**0.010797764**	**Trap**
**Energy**	**-0.207879234**	**0.038572756**	**-5.38927614**	**8.67E-08**	**Trap**
**Liveliness**	**-0.07472857**	**0.027319278**	**-2.735378696**	**0.006332348**	**Trap**
**Speechiness**	**-0.115709017**	**0.025945026**	**-4.459776576**	**9.06E-06**	**Trap**

Supporting figures

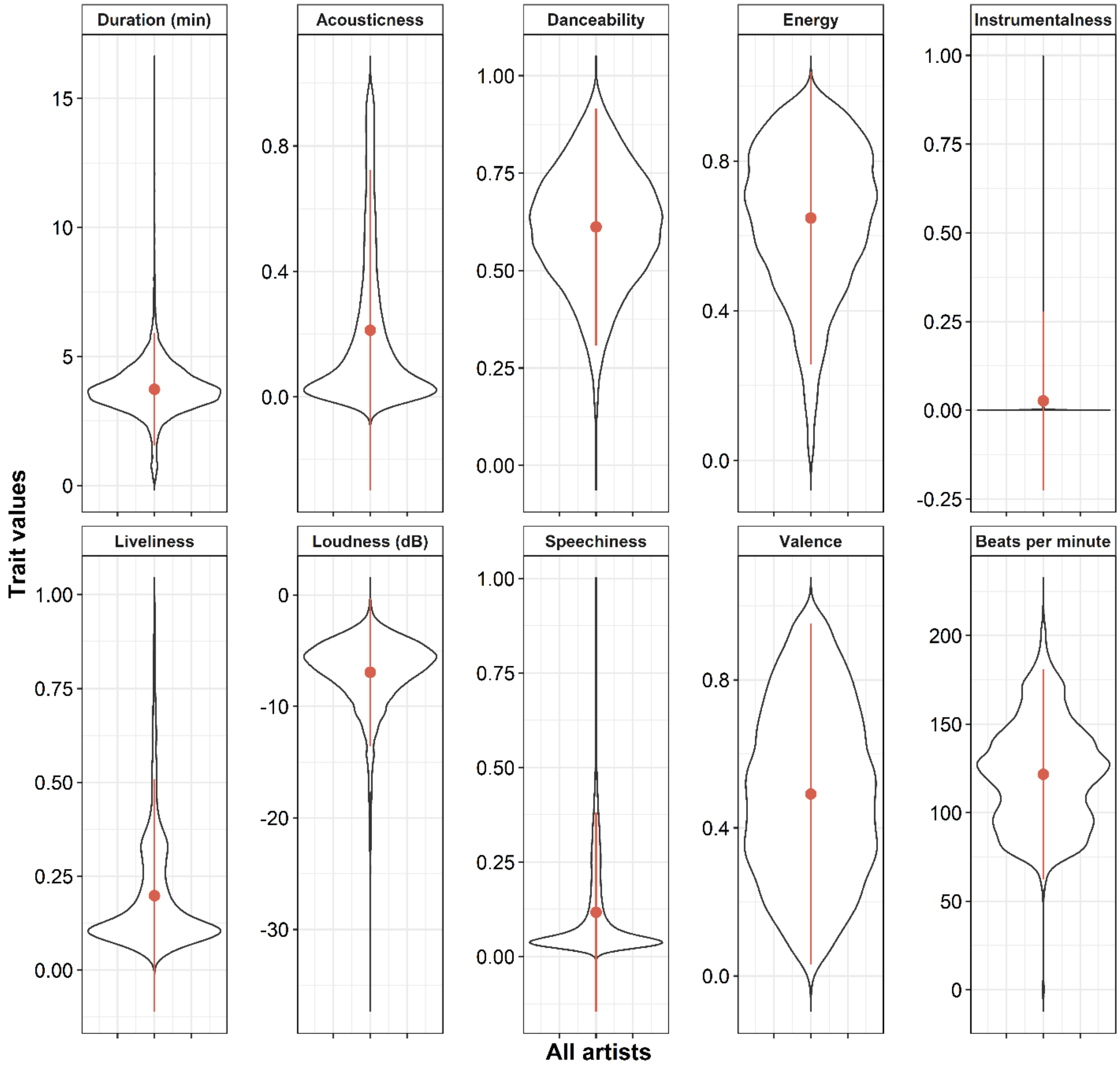

Fig S1. Distribution of continuous traits. Distribution of all ten continuous functional traits we retrieved in this study across all 10,444 songs from the 100 artists. Duration is presented in minutes, loudness is presented in decibels and beats per minute is presented in number of beats per minute, whereas all other traits are presented as an index that ranges from 0 to 1.

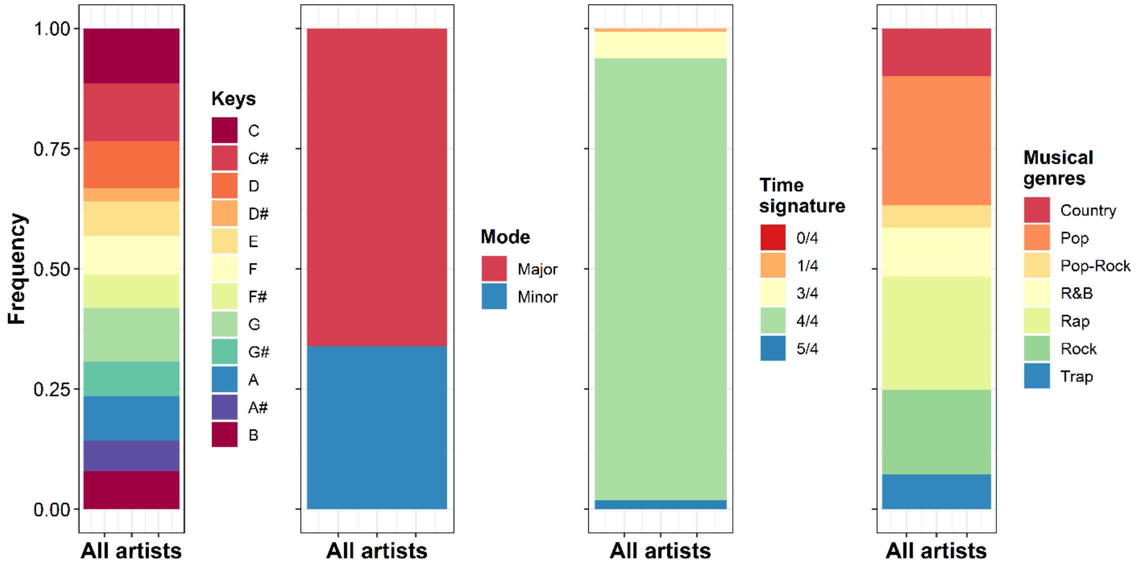

Fig S2. Frequency of categorical traits. Frequency of all categorical (i.e., key, mode and time signature) and multichoice traits (i.e., musical genres) we retrieved in this study across all 10,444 songs from the 100 artists.

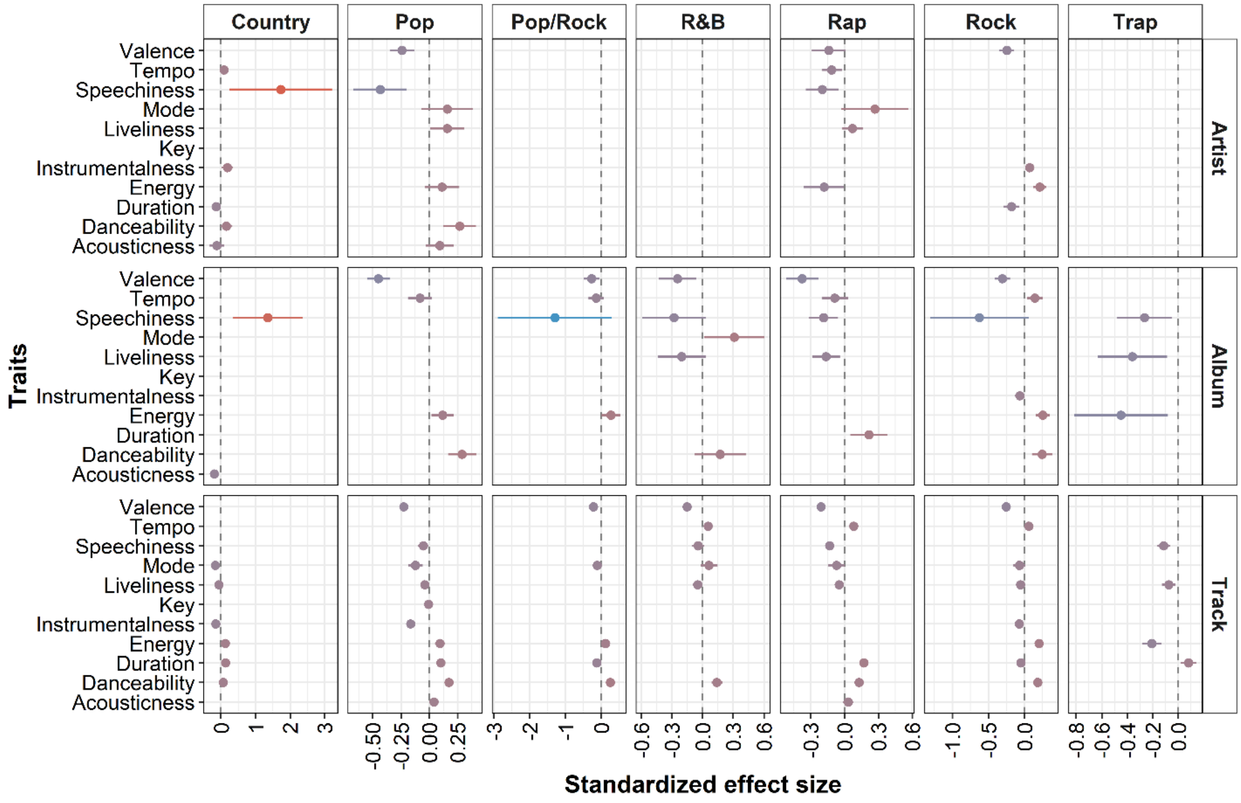

Fig S3. Associations between popularity and traits per musical genre. Associations between popularity and traits per musical genre. Standardized effect size of the association between the selected traits and the number of times that artists (top panels), their albums (middle panels), and songs (bottom panels) were played in Spotify by musical genre. Whiskers represent the 95% confidence interval of the mean estimate, which is represented by the points. Color of whiskers represents the size of the effect in the negative direction (blue whiskers) and the positive direction (red whiskers). Traits that do not have a whisker in a plot were not selected in the linear model after stepwise regression (selected models in S10 Table).

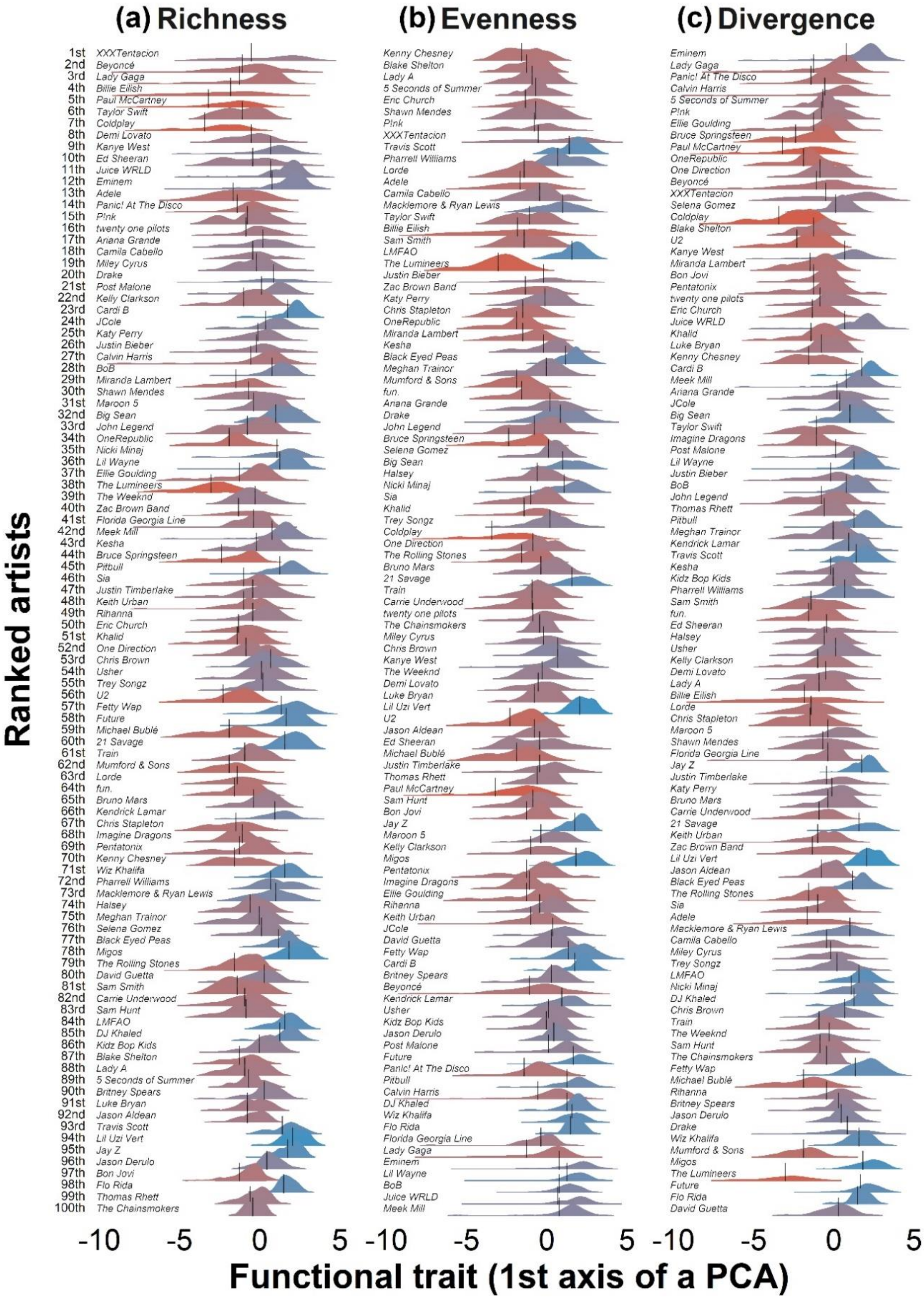
(a) Richness
(b) Evenness
(c) Divergence
Ranked artists
1st XXXTentacion
2nd Beyoncé
3rd Lady Gaga
4th Billie Eilish
5th Paul McCartney
6th Taylor Swift
7th Coldplay
8th Demi Lovato
9th Kanye West
10th Ed Sheeran
11th Juice WRLD
12th Eminem
13th Adele
14th Panic! At The Disco
15th P!nk
16th twenty one pilots
17th Ariana Grande
18th Camila Cabello
19th Miley Cyrus
20th Drake
21st Post Malone
22nd Kelly Clarkson
23rd Cardi B
24th JCole
25th Katy Perry
26th Justin Bieber
27th Calvin Harris
28th BoB
29th Miranda Lambert
30th Shawn Mendes
31st Maroon 5
32nd Big Sean
33rd John Legend
34th OneRepublic
35th Nicki Minaj
36th Lil Wayne
37th Ellie Goulding
38th The Lumineers
39th The Weeknd
40th Zac Brown Band
41st Florida Georgia Line
42nd Meek Mill
43rd Kesha
44th Bruce Springsteen
45th Pitbull
46th Sia
47th Justin Timberlake
48th Keith Urban
49th Rihanna
50th Eric Church
51st Khalid
52nd One Direction
53rd Chris Brown
54th Usher
55th Trey Songz
56th U2
57th Fetty Wap
58th Future
59th Michael Bublé
60th 21 Savage
61st Train
62nd Mumford & Sons
63rd Lorde
64th fun.
65th Bruno Mars
66th Kendrick Lamar
67th Chris Stapleton
68th Imagine Dragons
69th Pentatonix
70th Kenny Chesney
71st Wiz Khalifa
72nd Pharrell Williams
73rd Macklemore & Ryan Lewis
74th Halsey
75th Meghan Trainor
76th Selena Gomez
77th Black Eyed Peas
78th Migos
79th The Rolling Stones
80th David Guetta
81st Sam Smith
82nd Carrie Underwood
83rd Sam Hunt
84th LMFAO
85th DJ Khaled
86th Kidz Bop Kids
87th Blake Shelton
88th Lady A
89th 5 Seconds of Summer
90th Britney Spears
91st Luke Bryan
92nd Jason Aldean
93rd Travis Scott
94th Lil Uzi Vert
95th Jay Z
96th Jason Derulo
97th Bon Jovi
98th Flo Rida
99th Thomas Rhett
100th The Chainsmokers
Kenny Chesney
Blake Shelton
Lady A
5 Seconds of Summer
Eric Church
Shawn Mendes
P!nk
XXXTentacion
Travis Scott
Pharrell Williams
Lorde
Adele
Camila Cabello
Macklemore & Ryan Lewis
Taylor Swift
Billie Eilish
Sam Smith
LMFAO
The Lumineers
Justin Bieber
Zac Brown Band
Katy Perry
Chris Stapleton
OneRepublic
Miranda Lambert
Kesha
Black Eyed Peas
Meghan Trainor
Mumford & Sons
fun.
Ariana Grande
Drake
John Legend
Bruce Springsteen
Selena Gomez
Big Sean
Halsey
Nicki Minaj
Sia
Khalid
Trey Songz
Coldplay
One Direction
The Rolling Stones
Bruno Mars
21 Savage
Train
Carrie Underwood
twenty one pilots
The Chainsmokers
Miley Cyrus
Chris Brown
Kanye West
The Weeknd
Demi Lovato
Luke Bryan
Lil Uzi Vert
U2
Jason Aldean
Ed Sheeran
Michael Bublé
Justin Timberlake
Thomas Rhett
Paul McCartney
Sam Hunt
Bon Jovi
Jay Z
Maroon 5
Kelly Clarkson
Migos
Pentatonix
Imagine Dragons
Ellie Goulding
Rihanna
Keith Urban
JCole
David Guetta
Fetty Wap
Cardi B
Britney Spears
Beyoncé
Kendrick Lamar
Usher
Kidz Bop Kids
Jason Derulo
Post Malone
Future
Panic! At The Disco
Pitbull
Calvin Harris
DJ Khaled
Wiz Khalifa
Flo Rida
Florida Georgia Line
Lady Gaga
Eminem
Lil Wayne
BoB
Juice WRLD
Meek Mill
Eminem
Lady Gaga
Panic! At The Disco
Calvin Harris
5 Seconds of Summer
P!nk
Ellie Goulding
Bruce Springsteen
Paul McCartney
OneRepublic
One Direction
Beyoncé
XXXTentacion
Selena Gomez
Coldplay
Blake Shelton
U2
Kanye West
Miranda Lambert
Bon Jovi
Pentatonix
twenty one pilots
Eric Church
Juice WRLD
Khalid
Luke Bryan
Kenny Chesney
Cardi B
Meek Mill
Ariana Grande
JCole
Big Sean
Taylor Swift
Imagine Dragons
Post Malone
Lil Wayne
Justin Bieber
BoB
John Legend
Thomas Rhett
Pitbull
Meghan Trainor
Kendrick Lamar
Travis Scott
Kesha
Kidz Bop Kids
Pharrell Williams
Sam Smith
fun.
Ed Sheeran
Halsey
Usher
Kelly Clarkson
Demi Lovato
Lady A
Billie Eilish
Lorde
Chris Stapleton
Maroon 5
Shawn Mendes
Florida Georgia Line
Jay Z
Justin Timberlake
Katy Perry
Bruno Mars
Carrie Underwood
21 Savage
Keith Urban
Zac Brown Band
Lil Uzi Vert
Jason Aldean
Black Eyed Peas
The Rolling Stones
Sia
Adele
Macklemore & Ryan Lewis
Camila Cabello
Miley Cyrus
Trey Songz
LMFAO
Nicki Minaj
DJ Khaled
Chris Brown
Train
The Weeknd
Sam Hunt
The Chainsmokers
Fetty Wap
Michael Bublé
Rihanna
Britney Spears
Jason Derulo
Drake
Wiz Khalifa
Mumford & Sons
Migos
The Lumineers
Future
Flo Rida
David Guetta
-10 -5 0 5 -10 -5 0 5 -10 -5 0 5
Functional trait (1st axis of a PCA)

Fig S4. Unidimensional functional space of artists. Unidimensional functional space of the characteristics of the songs for all artists ranked by their functional (a) richness, (b) evenness, and (c) divergence. Y axis represents the probability of the artists having songs in that part of the functional space. Lines in the middle of polygons indicate the centroid distribution of the traits of artists. Colors indicate trait values of artists in a way that blueish colors represent artists that released songs that are more danceable, energetic and rap, while reddish colors represent artists who released song that are more acoustic and rock-oriented.

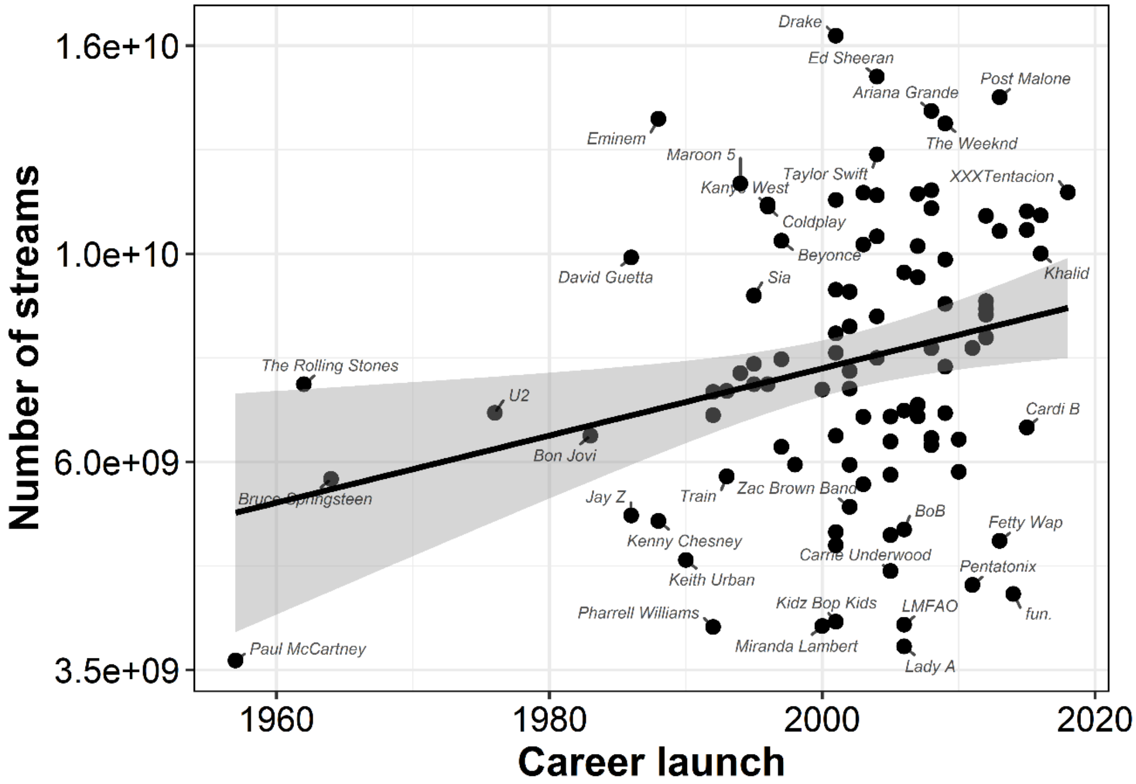

Fig S5. Association between year of career launch and popularity. Association between year of career launch and the artists' popularity (as total number of streams on Spotify). Total number of streams is represented in a logarithm scale for better visualization of data distribution. Lines represent the direction of significant associations between the two variables and the shaded area represent its 95% confidence interval.
